# Supplementary material for: Zinc-binding to the cytoplasmic PAS domain regulates the essential WalK histidine kinase of Staphylococcus aureus
Source: Nat Commun. 2019 Jul 11;10:3067. doi: 10.1038/s41467-019-10932-4 (PMC6624279; doi:10.1038/s41467-019-10932-4)
Supplement: Supplementary file 1 — Supplementary Information [file 41467_2019_10932_MOESM1_ESM.pdf]

SUPPLEMENTARY INFORMATION

Zinc-binding to the cytoplasmic PAS domain regulates the essential WalK histidine kinase of *Staphylococcus aureus*

Monk *et al.*

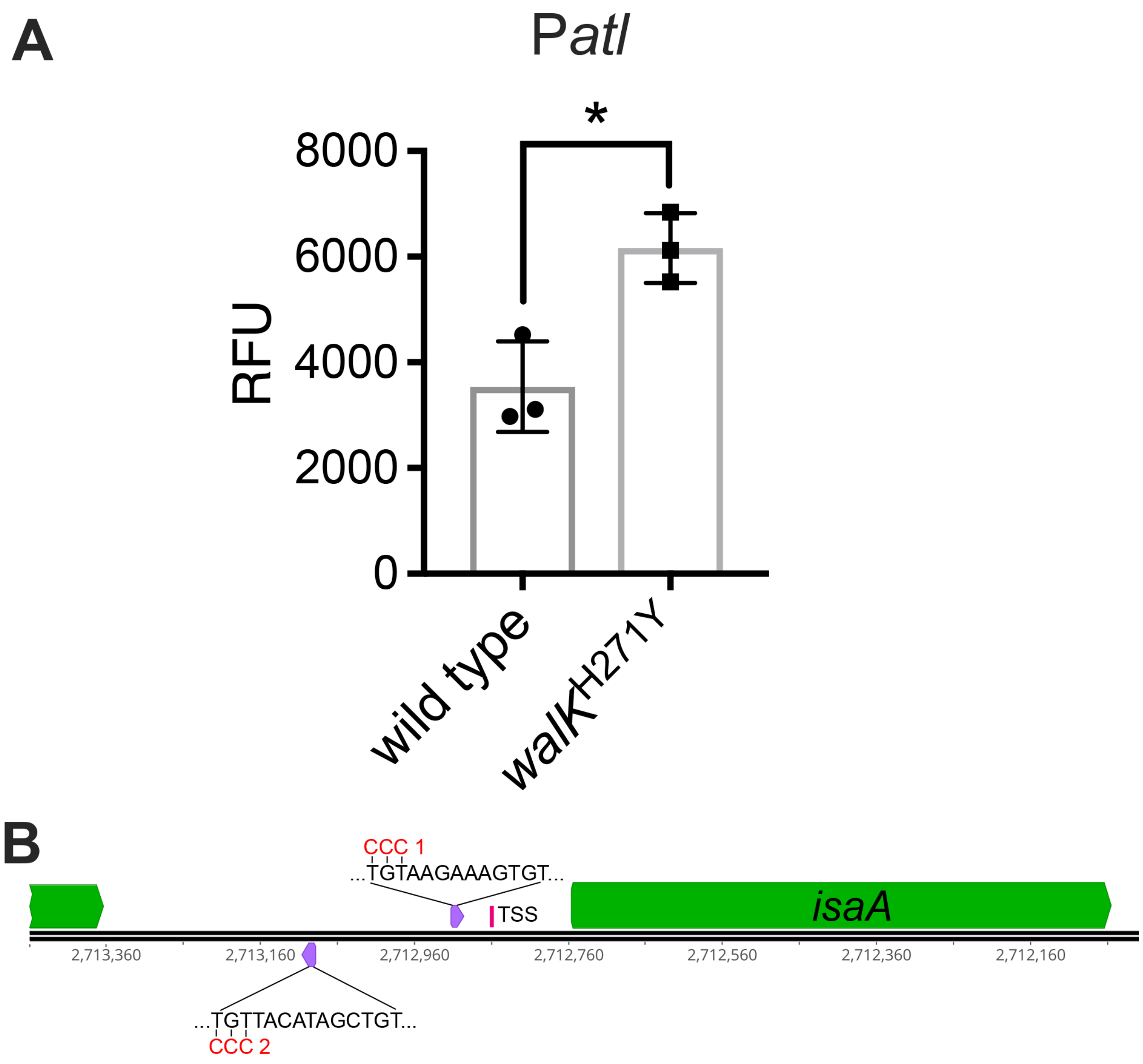

Supplementary Figure 1. Expression of P*atl* is upregulated in the NRS384 *walK*^{H271Y} mutant. **(A)** Assessment of the impact of the *walK*^{H271Y} mutation on expression of the major *S. aureus* autolysin encoded by *atl*. The promoter region of *atl* was transcriptionally fused with the enhanced YFP reporter plasmid and transformed into NRS384 and NRS384 *walK*^{H271Y}. Null hypothesis (no difference between means) rejected for $p<0.05$ (Unpaired, 2-tailed, Student's t test). **(B)** Overview of the *isaA* promoter region, showing the location, and sequence of the two WalR-binding motifs and the conserved 'TGT' motif mutated to 'CCC' (refer Figure 4e).

A

Amino acids 251-376 of WalK:

1-VQEAQANTESEKRRLDSVITHMSDGIIATDRRGRIRIVNDMAL
KMLGMAKEDIIGYYMLSVLSLEDEFKLEEIQENNDSFLLDLNEEE
GLIARVNFSTIVQETGFVTGYIAVLHDVTEQQQVERER-126

B

Mass	Corrected mass	Position	#MC	Peptide sequence	MALDI
6292.1207		70-124	1	LEEIQENNDSFLLDLNEEEG LIARVNFSTIVQETGFVTGY IAVLHDVTEQQQVER	No
5048.4496	5095.4496	51-93	1	EDIIGYYsMLSVLSLEDEFKL EEIQENNDSFLLDLNEEEGL IAR	No
3792.9191		94-126	1	VNFSTIVQETGFVTGYIAVL HDVTEQQQVERER	No
3507.7754		94-124	0	VNFSTIVQETGFVTGYIAVL HDVTEQQQVER	Yes
2895.4228	3036.4228	45-69	1	sMLGsMAKEDIIGYYsMLSVLSL EDEFK	No
2803.3632		70-93	0	LEEIQENNDSFLLDLNEEEG LIAR	No
2264.1042	**2311.1042**	51-69	0	EDIIGYYsMLSVLSLEDEFK	Yes
2000.0229	**2047.0229**	14-31	1	RLDSVITHsMSDGIIATDR	Yes
2000.0229	**2047.0229**	15-32	1	LDSVITHsMSDGIIATDRR	Yes
1843.9218	**1890.9218**	15-31	0	LDSVITHsMSDGIIATDR	Yes
1534.8154	**1675.8154**	37-50	1	IVNDsMALKsMLGsMAK	Yes
1489.7241		1-13	1	VQEAQANTESEKR	No
1333.623		1-12	0	VQEAQANTESEK	No
1172.682	1219.682	35-44	1	IRIVNDsMALK	No
903.4968	950.4968	37-44	0	IVNDsMALK	No
650.3364	744.3364	45-50	0	sMLGsMAK	No
501.3256		33-36	1	GRIR	No

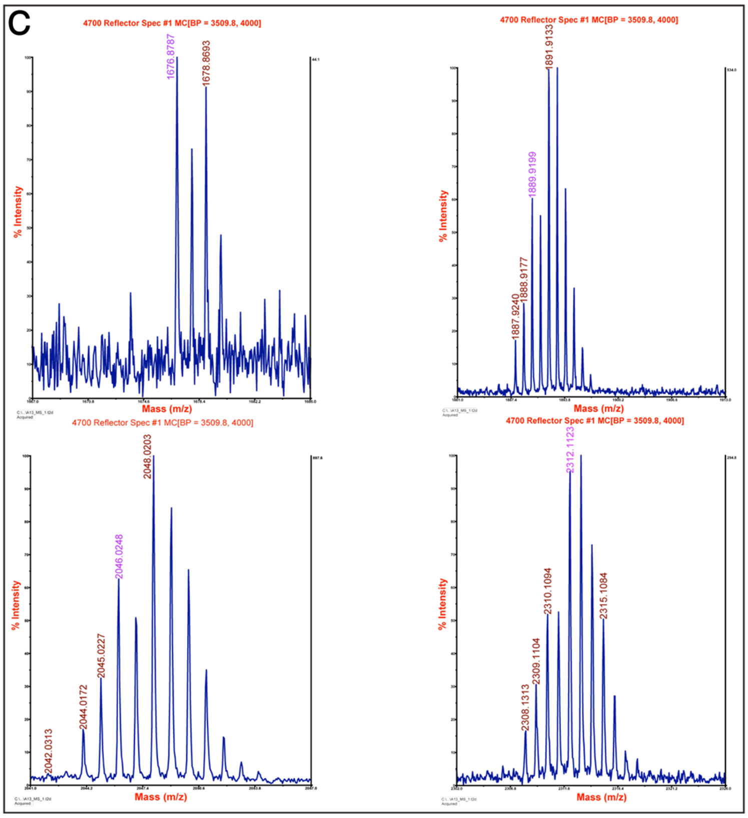

Supplementary Figure 2. Incorporation of selenomethionine in WalK-PAS^{FULL} and detection by MALDI-MS. (A) Amino acids from 251-376 of the WalK-PAS^{FULL} construct with methionine residues highlighted in red. **(B)** Peptide fragmentation prediction for WalK-PAS^{FULL} for trypsin digestion and the detected fragments. The highlighted corrected masses were detected using MALDI-MS. **(C)** MALDI spectra of tryptic fragments showing the corrected mass (highlighted in purple). The molecular weight difference in the individual fragmented peptide corresponded to the number of selenomethionines in the peptide. The mass analysis verified the 100% substitution of SeMet in WalK-PAS^{FULL} domain.

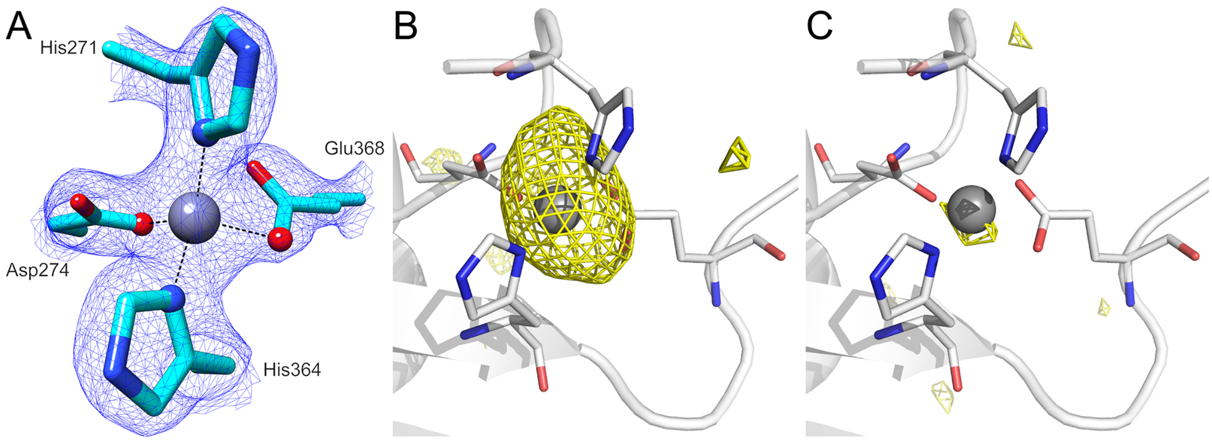

Supplementary Figure 3. Confirmation of the metal identity in the Zn^{2+} binding site in WalK-PAS^{FULL}. (A) The Zn^{2+}-coordinating residues of WalK-PAS^{FULL} are shown as cyan sticks, with the atoms contributing to the interactions as spheres. The coordinating bonds are illustrated with black dashed lines. The electron density shown is the $2F_o$-F_c map contoured at 1.5 σ. **(B)** The Zn^{2+}-binding site of WalK-PAS^{FULL} is shown, with metal-coordinating residues as white sticks and the Zn atom as a gray sphere. Anomalous difference Fourier maps at 1.26 Å (Zn edge) and **(C)** 1.31 Å (low energy of Zn edge) wavelengths are shown in yellow. Both maps are contoured at 3σ. A peak at ~10σ is observed at 1.26 Å (Zn f"~3.8 e^-), coincident with the modelled position of the Zn atom. This peak decreases to < 3σ at 1.31 Å (Zn f"~0.5 e^-), confirming the identity of the bound metal as Zn.

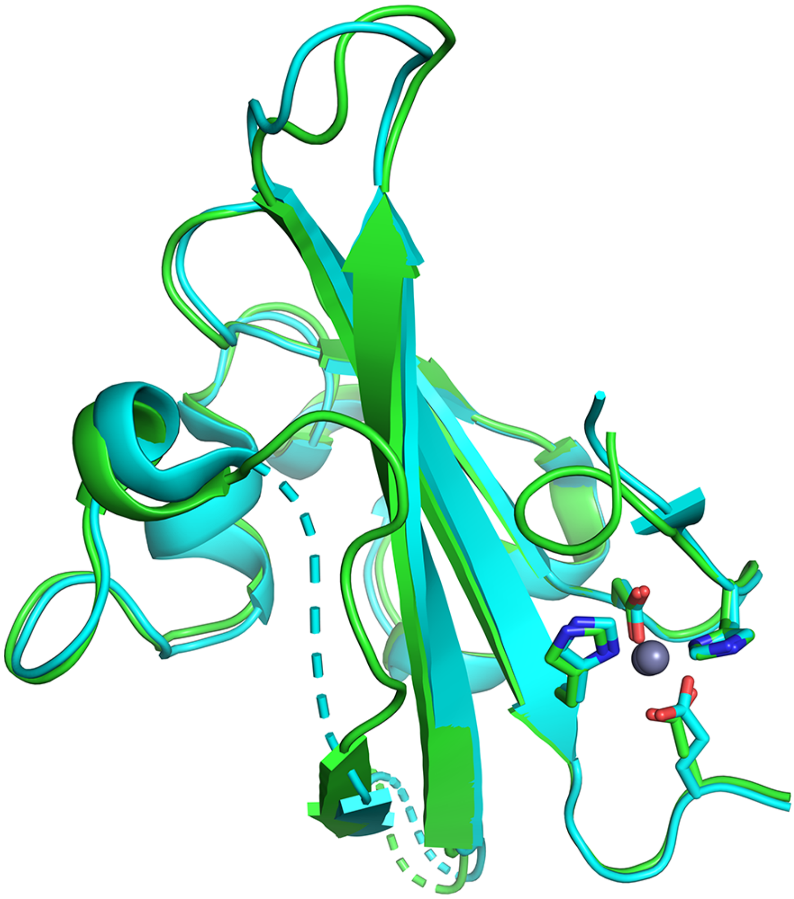

Supplementary Figure 4. Structural comparison of WalK-PAS^{FULL} with WalK-PAS^{TRUNC}. Superposition of the crystal structure of WalK-PAS^{FULL} (green) with WalK-PAS^{TRUNC} (light blue) in cartoon representation. The bound Zn^{2+} ions are shown as spheres and the Zn^{2+}-coordinating residues are shown as sticks.

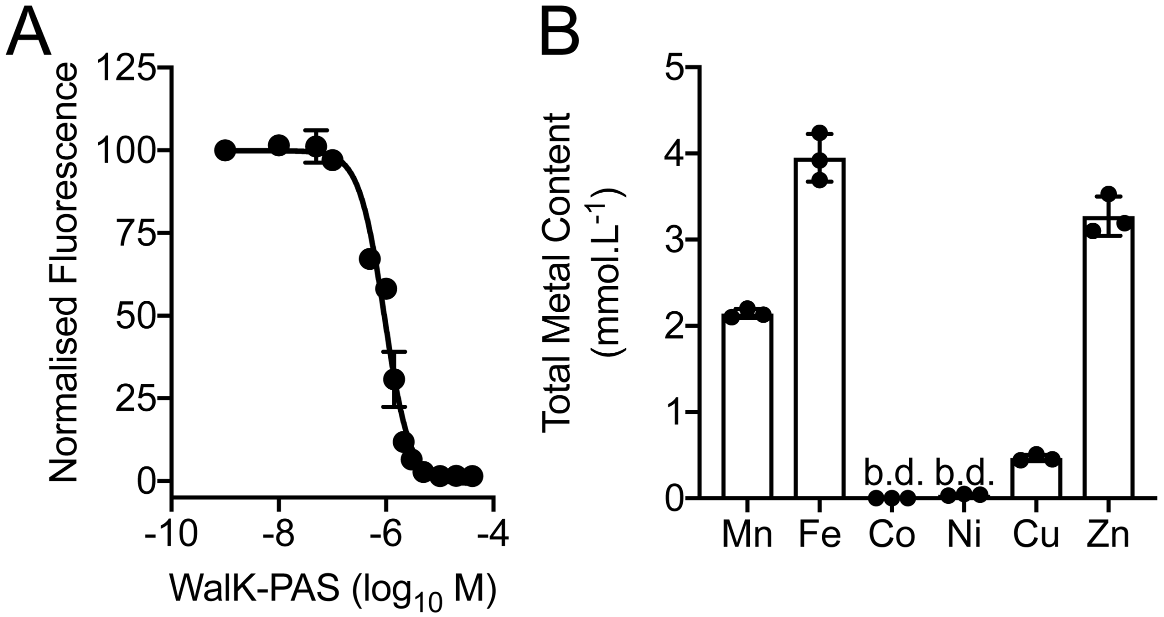

Supplementary Figure 5. WalK-PAS affinity determination and cellular abundance of Zn^{2+} ions. (**A**) Competitive Zn^{2+} binding by apo-WalK-PAS^{FULL} from FluoZin-3-Zn^{2+}. Apo-WalK-PAS^{FULL} was titrated against 150 nM FluoZin-3-Zn^{2+} until fluorescence was quenched. The fluorescence data was normalized using experimentally observed fluorescence minimum and maximum values. Each data point corresponds to the mean (± s.e.m.) of six independent experiments. Source data are provided as a Source Data file. **(B)** Total cellular metal ion accumulation represented as the mean (± s.e.m.) concentration (mmol.L^{-1}) of ions per cell (determined as CFU) of known cell volume (determined by SEM). The values are from three independent biological experiments. Metal-ions assessed were Mn, Fe, Co^{2+}, Ni, Cu, and Zn, with b.d. denoting below detection limit.

>sp|Q2G2U4| OS=Staphylococcus aureus (strain NCTC 8325) OX=93061 GN=walK PE=1 SV=1

WALK_STAA8 Sensor protein kinase WalK

Homology modelling map

Sequence:	Structure overlap
MKWLKQLQSLHTKLVIVYVLLIIIGMQIIGLYFTNNLEKELLDNFKKNITQYAKQLEISI	
EKVYDEKGSVNAQKDIQNLLSEYANRQEIGEIRFIDKDQIIIATTKQSNRSLINQKANDS	5IS1
SVQKALSLGQSNDHLILKDYGGGKDRVWVYNIPVKVDKKVIGNIYIESKINDVYNQLNNI	
NQIFIVGTAISLLITVILGFFIARTITKPITDMRNQTVEMSRGNYTQRVKIYGNDEIGEL	
ALAFNNLSKRVQEAQANTESEKRRLDSVITHMSDGIIATDRRGRIRIVNDMALKMLGMAK	
EDIIGYYMLSVLSLEDEFKLEEIQENNDSFLLDLNEEEGLIARVNFSTIVQETGFVTGYI	4MN6
AVLHDVTEQQQVERERREFVANVSHELRTPLTSMNSYIEALEEGAWKDEELAPQFLSVTR	
EETERMIRLVNDLLQLSKMDNESDQINKEIIDFNMFINKIINRHEMSAKDTTFIRDIPKK	4I5S
TIFTEFDPDKMTQVFDNVITNAMKYSRGDKRVEFHVKQNPLYNRMTIRIKDNGIGIPINK	
VDKIFDRFYRVDKARTRKMGGTGLGLAISKEIVEAHNGRIWANSVEGQGTSIFITLPCEV	
IEDGDWDE	

Supplementary Figure 6. Homology modelling map of pdb structures used to build the *S. aureus* WalK model. Red region corresponds to structure pdb 5IS1 (residues 33-182, identity 100%), orange to pdb structure 4MN6 (residues 266 to 371, identity 100%), and underlined to pdb structure 4I5S (residues 223 to 599, identity 44%). Blue regions did not have significant similarities to the pdb. The N-terminus (1-32) was modelled as transmembrane helix, as was the middle sequence (183- 222). The C-terminus was modelled as disordered.

Supplementary Table 1: Diffraction data collection and refinement statistics for WalK-PAS^{FULL} and WalK-PAS^{TRUNC}.

	**Selenomethionine WalK-PAS^{FULL}**	**WalK-PAS^{FULL}**	**WalK-PAS^{TRUNC}**	**WalK-PAS^{FULL} Zn peak edge**	**WalK-PAS^{FULL} Zn low energy edge**
**Data Collection**					
**Wavelength (Å)**	0.9537	0.918	0.9537	1.26	1.31
**Space group**	$P2_12_12_1$	$P2_12_12_1$	$P2_12_12_1$	$P2_12_12_1$	$P2_12_12_1$
**Unit cell (Å)**					
***a, b, c* (Å)**	57.8, 60.9, 84.1	58.8, 60.5, 84.5	43.8, 61.2, 95.6	46.5, 55.3, 93.3	46.6, 55.4, 93.5
***α, β, γ* (°)**	90, 90, 90	90, 90, 90	90, 90, 90	90, 90, 90	90, 90, 90
**Resolution (Å)**	57.8–2.50 (2.64–2.50)	42.25–2.00 (2.11–2.00)	51.5–2.10 (2.21–2.10)	47.6–2.79 (2.95–2.79)	47.7–2.99 (3.17–2.99)
**Unique reflections**	10780 (1554)	20961 (3009)	214422 (15549)	80254 (6357)	65457 (10421)
**Multiplicity**	13.4 (13.9)	9.3 (9.5)	13.8 (14.3)	12.6 (13.0)	12.5 (12.9)
**Completeness (%)**	100.0 (100.0)	100.0 (100.0)	99.9 (100.0)	99.7 (98.3)	99.6 (97.7)
**Anomalous multiplicity**	7.3 (7.3)	5.0 (4.9)	-	7.0 (7.0)	6.9 (7.1)
**Anomalous completeness**	100.0 (100.0)	99.7 (99.7)		99.6 (97.2)	99.2 (95.2)
**R_{merge}**	0.091 (0.944)	0.070 (0.742)	0.08 (1.13)	0.09 (2.60)	0.07 (1.38)
**I/σ(I)**	17.4 (2.8)	15.8 (2.5)	17.5 (2.4)	17.5 (2.4)	14.7 (1.0)
**Phasing**					
**Figure of merit**	0.48				
**Number of sites**	19				
**Bayes correlation coefficient**	56.9 ± 7.2				
**Refinement**					
**Resolution (Å)**		34.60–2.00 (2.09–2.00)	47.82-2.10 (2.23–2.10)		
**Number of reflections**		20907 (2432)	15502(2393)		
**R_{free}**		1072 (146)	777 (123)		
**R_{work}/R_{free}**		0.222 (0.286)/0.253 (0.357)	0.225 (0.276)/0.274 (0.309)		
**Number of atoms**					
**Protein**		1631	1500		
**Solvent**		76	53		
**Zinc**		2	2		
**Average *B*-factor (Å^2)**		48.4	69.4		

**Average *B*-factor Zinc (Å^2)**	41.0	50.2
**R.m.s deviations**		
**Bond lengths (Å)**	0.008	0.008
**Bond angles (°)**	1.07	0.48
**Clashscore**	4.0	12
**Ramachandran allowed/disallowed (%)**	100/0	99.4/0.6
**PDB code**	6MN5	6MN6

Statistics for the highest-resolution shell are shown in parentheses.

Supplementary Table 2: Strains and plasmids used in this study

***Escherichia coli* strains**	**Description**	**Reference**
DH5 alpha	K-12 cloning strain of *E. coli*	Thermo Fisher
BL21(DE3)	B-strain. F$^-$ *ompT hsdS*$_B$ (r_B^- m_B^-) *gal dcm* (DE3); IPTG-inducible T7 RNA polymerase.	Novagen
IM08B	DH10BΔ*dcm.* Expresses CC8 adenine methylation profile.	[1]
***Staphylococcus aureus* strains**		
NRS384	USA300-14 clone. Tetracycline resistant	BEI resources
NRS384 *walK*^{H271Y}	Introduction of the WalK^{H271Y} mutation in PAS domain. Upregulates WalK activity. No longer binds zinc.	This study
NRS384 *walK*^{H271Y}complemented	Reversion of the WalK^{H271Y} mutation, introduction of a silent PstI site into the gene.	This study
NRS384 Δ*yycHI*	Deletion of WalKR positive regulators *yycHI.* Clean deletion from codon 5 of *yycH* to the TAA of *yycI*	This study
NRS384 Δ*yycHI walK*^{H271Y}	Purified sectored outgrowth of *ΔyycHI.* Spontaneous acquisition of *walK*^{H271Y}mutation	This study
NRS384 *walK*^{G223D}	WalK^{G223D} mutation in HAMP domain introduced. Down regulates WalK activity.	This study
NRS384 Δ*atl*	Deletion of *atl.* Peptidoglycan hydrolase regulated by WalKR.	This study
NRS384 *walR*^{FLAG}	3xFLAG tag introduced on the C-terminus of WalR in the wild type strain.	This study
NRS384 *walK*^{H271Y} *walR*^{FLAG}	3xFLAG tag introduced on the C-terminus of WalR in the *walK*^{H271Y} background.	This study

NRS384 Δ*stp1*	Deletion of the serine threonine phosphatase gene, from the ATG leaving the last 8 amino acids.	This study
NRS384 Δ*pknB*	Deletion of the serine threonine kinase gene, from the ATG to the TAA.	This study
Plasmids		
pIMAY-Z	Allelic exchange plasmid.	[1]
pRAB11	Tetracycline inducible expression plasmid.	[2]
pGEX-2T	Protein expression plasmid. Introduces a N-terminal GST tag.	GE healthcare
pET19b	Protein expression plasmid. Introduces a N-terminal His_6 tag.	Novagen
pIMC8-YFP	Enhanced YFP reporter plasmid.	This study
pIMAY-Z *walK*^{H271Y}	Full length WalK containing the H271Y mutation.	This study
pIMAY-Z *walK*^{PstI}	Full length WalK with a silent PstI site introduced 1302 nt.	This study
pIMAY-Z Δ*yycHI*	Deletion of *yycHI* from the 5th codon of *yycH* and stop codon of *yycI*.	This study
pIMAY-Z Δ*atl*	Deletion of *atl* from the ATG to the TAA of *atl*.	This study
pIMAY-Z *walR*^{FLAG}	Introduction of the 3x-FLAG tag on to the C terminus of WalR.	This study
pIMAY-Z Δ*stp1*	Deletion of *stp1,* from the ATG leaving the last 8 codons.	This study
pIMAY-Z Δ*pknB*	Deletion of *pknB*, from the ATG to the TAA of *pknB*.	This study
pRAB11 *walR*^{FLAG}	Construct for Tetracycline inducible WalR^{FLAG} (3x FLAG on the C-terminus).	This study
pRAB11 *walR*$^{D53A\text{-}FLAG}$	Construct for Tetracycline inducible WalR^{FLAG} with the phosphorylated aspartic acid changed to alanine.	This study
pIMAY-Z *walK*^{G223D}	Full length WalK gene from JKD6009 containing the G223D mutation.	This study
pGEX-2T(WalK-PAS^{FULL})	Full length PAS domain as defined by limited proteolysis (251-376 aa) with a N-terminal GST tag.	This study
pGEX-2T(WalK-PAS^{TRUNC})	Reduced PAS domain (266-371 aa) with a N-terminal GST tag.	This study
pGEX-2T(WalK-PAS$^{TRUNC\ H271Y}$)	Reduced PAS domain (266-371 aa) with a N-terminal GST tag containing the H271Y change.	This study
pET19b(WalK^{CYT})	Cytoplasmic domains of WalK (208-608 aa) with an N-termianl 6xHIS tag.	This study
pET19b(WalK$^{CYT\text{-}H271Y}$)	Cytoplasmic domains of WalK (208-608 aa) containing the H271Y mutation with an N-terminal His_6 tag.	This study
pIMC8-P*isaA*-YFP	Native *isaA* driven expression of YFP	This study
pIMC8-P*isaA*CCCI-YFP	*isaA* driven expression of YFP. Mutation in the WalR binding site 48 nt upstream from the TSS	This study

pIMC8-P*isaA*CCCII-YFP	*isaA* driven expression of YFP. Mutation in the WalR binding site 228 nt upstream from the TSS	This study
pIMC8-P*atl*-YFP	*atl* driven expression of YFP.	This study

Supplementary Table 3: Primers used in this study

**walKH271Y, complementation and walKG223D**	**Oligonucleotide 5'-3'**	**Restriction site**
IM7 H271Y AF	**CCTCACTAAAGGGAACAAAAGCTGGGTACC**AGGTCGAAACGAATGAAGTGGCTAAAAC	
IM8 H271Y BR	atGTGATAACTGAGTCCAGTCTACGTTTCTCAC	
IM9 H271Y CF	AGAAACGTAGACTGGACTCAGTTATCACatATATGAGTGATGGTATTATTGCAACAGACC	
IM10 H271Y DR	**CGACTCACTATAGGGCGAATTGGAGCTC**CTCCTTATTATTCATCCCAATCACCGTC	
IM11 H271Y screen F	TATGACGAAAAGGGCTCCGTAAATGC	
IM12 H271Y screen R	TGTTGCAATAATACCATCACTCATATat	
IM58 walK PstI+ BR	cAGCAAGTCATTGACCAGTCGAATC	
IM59 walK PstI+ CF	GAATGATTCGACTGGTCAATGACTTGCTGcAG	
**atl deletion**		
IM96 Atl AF	**CCTCACTAAAGGGAACAAAAGCTGGGTACC**AATCTGAATCTATTCACCTCATTGG	
IM97 Atl BR	CATTCTATTTATTACTCCTAACATTTAT	
IM98 Atl CF	*ATAAATGTTAGGAGTAATAAATAGAATG*TAAGCAACATGAACATAGGATCAAAAG	
IM99 Atl DR	**CGACTCACTATAGGGCGAATTGGAGCTC**AAATCTGAACAGCTAGAACTTCTCC	
IM100 Atl OUT F	TGTAGAAGAACAAGGCGTCCCTGAGG	
IM101 Atl OUT R	TGAATAGAAGATTTGGACAACGCACG	
**yycHI deletion**		
IM54 yycH AF	**CCTCACTAAAGGGAACAAAAGCTGGGTACC**GACTTGCTACAGTTATCTAAAATGG	
IM78 yycH BR	CTCCTTATTATTCATCCCAATCACCGTC	
IM79 yycHI CF	*CGGTGATTGGGATGAATAATAAGGAG*TAATATGAATCGTAATAAGCTAGCATTGC	
IM75 yycI DR	**CGACTCACTATAGGGCGAATTGGAGCTC**TCTTCCATTTTCTTTCCAGTCAAACC	
IM80 yycH OUT F	AGAAACAGAACGAATGATTCGACTGG	

IM77 yycI OUT R	CGCATAAATTGGCAATTGATATTTACG	
**Stp1/PknB deletion**		
IM251 stp1 A	**CCTCACTAAAGGGAACAAAAGCTGGGTACC**ACGTCACATTACAGTATCAACATCAGG	
IM252 stp1 B	CATTTGTCTTTACCTCGTTTCTACTTGTCG	
IM253 stp1 C	*ACAAGTAGAAACGAGGTAAAGACAAATG*GCGGCTATTGAAGGTGATAAAGTatgATAGG	
IM254 stp1 D	**CGACTCACTATAGGGCGAATTGGAGCTC**TGGCGAAAAGTACTGCACAGTACC	
IM255 pknB A	**CCTCACTAAAGGGAACAAAAGCTGGGTACC**ATGAGTTGAAATCCCGTTTTGAAGC	
IM256 pknB B	CATACTTTATCACCTTCAATAGCCGCGAG	
IM257 pknB C	*CGGCTATTGAAGGTGATAAAGTATG*TAAATATAATTGAAGTAAATGTACCGAGG	
IM258 pknB D	**CGACTCACTATAGGGCGAATTGGAGCTC**TCGCATTTAACTGATACGAATGTGC	
**WalR-FLAG insertion**		
IM31 walR AF	**CCTCACTAAAGGGAACAAAAGCTGGGTACC**CGTCCATTTTCTTTAAAATGTATGAACC	
IM108 walR FLAG BR	*ATCTTTATAATCTTTGTCATCATCATCTTTGTAATC*CTCATGTTGTTGGAGGAAATATCC	
IM109 walR FLAG CF	**GATTACAAAGATGATGATGACAAAGATTATAAAGATGACGACGATAAAGACTACAAGGATGATGACGATAAA**TAGAGGTCGAAACGAATGAAGTGG	
IM40 walR DR	**CGACTCACTATAGGGCGAATTGGAGCTC**TGGGATATTATATACCCAGACACGGTCC	
IM111 walR screen F	GTTTACGTGAAAAGATTGAAGATG	
IM181 walR screen R	TTTTTCAAGGTTATTTGTAAAATATAACCC	
**pRAB11 walR**		
IM280 walR(KpnI) AF	ATATGGTACCGGAGGAATTGGAAATGCAAATGGCTAGAAAAGTTGTTG	KpnI
IM278 walR D55A BR	cgCTAGTAATACGATGTCTGGTTCTTCTTC	
IM279 walR D55A CF	GAAGAACCAGACATCGTATTACTAGcgATCATGTTACCTGGTCGTGATGG	
IM305 walR (BamHI) DR	ATATGGATCCTTAGCCACTTCATTCGTTTCGACC	BamHI
WalK-PAS		
WalK 251-F	ATATGGATCCGTACAAGAAGCGCAGGCTAATAC	BamHI
WalK 376-R	ATATGAATTCACGCTCACGTTCAACTTGTTGTTG	EcoRI
WalK-CYTO		
WalK 208-F	GGAATTCCATATGAAACCAATCACCGATATGCGTAAC	NdeI
WalK TAA-R	CGGGATCCTTATTCATCCCAATCACCGTCTTC	BamHI

pIMC8-YFP		
IM385 pIMC8 INV F	TGATTAACTTTATAAGGAGGAAAAACATATG	
IM363 isaA F	**CGACGGTATCGATAAGCTTGATATC**TAACAGTATGTTTTTTGAAAATATGAGACC	
IM364 isaA R	**ATGTTTTTCCTCCTTATAAAGTTAATCA**AGTAAAAAATCCTCCAGTAATAATTG	
IM1122 2506 (ccc-1)F	ACACTTGATATTGTAATGTTTcccAAAGAAAGTGTAATTTACTGGCTGG	
IM1123 2506 (ccc-1)R	gggAAACATTACAATATCAAGTGTTATTTG	
1124 2506 (ccc-2)F	CAGATATATTACAGCTATGTAgggAAAATACAATCTGTAATATTACGAAAGC	
1125 2506 (ccc-2)R	cccTACATAGCTGTAATATATCTGACATGTAAC	

Notes: Bold = Complementary to pIMAY-Z or pIMC8-YFP for SLiCE cloning; Italics = Tail of C primer complementary to B primer for SOE-PCR; Underlined = Restriction site; Lowercase = mutated bases.

References:

1. Monk, I.R., et al., *Complete Bypass of Restriction Systems for Major Staphylococcus aureus Lineages.* mBio, 2015. **6**(3).
2. Helle, L., et al., *Vectors for improved Tet repressor-dependent gradual gene induction or silencing in Staphylococcus aureus.* Microbiology, 2011. **157**(Pt 12): p. 3314-23.
